# Invasive lobular breast cancer in young patients without adjuvant systemic treatment: 15-year survival outcomes

**DOI:** 10.1007/s10549-026-08024-1

**Published:** 2026-08-06

**Authors:** R. A. B. Voorthuis, M. Opdam, N. D. Ter Hoeve, M. da Silva, M. Christgen, G. Dackus, P. V. Diest, P. W. B. Derksen, F. S. Hilbers, S. C. Linn

**Affiliations:** 1 Division of Molecular Pathology, Netherlands Cancer Institute, Amsterdam, The Netherlands; 2 Department of Pathology, University Medical Center Utrecht, Utrecht, The Netherlands; 3 Department of Pathology, Netherlands Cancer Institute, Amsterdam, The Netherlands; 4 Institute of Pathology, Hannover Medical School, Hannover, Germany; 5 Department of Medical Oncology, Netherlands Cancer Institute, Amsterdam, The Netherlands

**Keywords:** Invasive lobular carcinoma, Prognosis, Prognostic factors, Young women, Under 40

## Abstract

**Purpose:**

Invasive lobular carcinoma (ILC) of the breast typically affects older women. As a result, there is limited research on prognosis and prognostic factors in young women with ILC. Here we set out to determine the long-term outcome and prognostic factors of women under 40 years of age with lymph node-negative (N0), hormone-receptor positive (HR+)/human epidermal growth-factor receptor 2 negative (HER2-) ILC who did not receive adjuvant systemic treatment (chemo- and/or endocrine therapy).

**Methods:**

Through the Netherlands Cancer Registry we selected all systemically untreated patients younger than 40 years with HR+/HER2-, N0, early breast cancer diagnosed in the Netherlands from 1989-2000. At the time, node-negativity was considered a favorable prognostic marker and guidelines recommended no adjuvant systemic therapy for this subgroup. Distant recurrence-free survival (DRFS), recurrence-free survival (RFS) and overall survival (OS) were assessed according to the STEEP criteria. Uni- and multivariable Cox proportional hazard models were used to assess prognosis, calculating hazard ratios (HR) with confidence interval (CI).

**Results:**

Of the *n* = 1138 selected patients, *n* = 95 were histologically classified as ILC and *n* = 805 as invasive breast cancer of no special type (BC-NST), *n* = 238 were classified as other breast cancer subtype. In ILC, tumor grade was not associated with DRFS/RFS or OS (HR for DRFS 1.40, 95%CI 0.42–4.63). However, the presence of lymphovascular invasion (LVI) (adjusted HR DRFS 2.81, 95%CI 1.14–6.94, *p* < 0.05) and multifocality (adjusted HR DRFS 3.28, 95%CI 1.49–7.22, *p* < 0.01) were. When comparing DRFS, RFS and OS in matched low-risk luminal A-like ILC vs. BC-NST, we observed a trend for worse 15-year survival in ILC (15-year DRFS 63% in ILC vs. 74% in BC-NST; HR 1.50, 95%CI 0.98–2.29, *p* = 0.06).

**Conclusion:**

In these young patients with systemically untreated N0 ILC we show that grade was not prognostic at 15 years follow-up, whereas LVI and multifocality were. Furthermore, ILC had a numerically worse 15-year survival than matched luminal A-like BC-NST.

**Supplementary Information:**

The online version contains supplementary material available at 10.1007/s10549-026-08024-1.

## Introduction

Invasive lobular carcinoma (ILC) is the second most prevalent histological breast cancer subtype responsible for 15% of all invasive breast cancers [1]. ILC is often diagnosed in older women, with a median age of 57–65 years [2]. As a result, there is limited research on prognosis and prognostic factors in young patients with ILC.

Non-hereditary ILC often harbors deleterious or inactivating mutations of the tumor-suppressor CDH1 gene which encodes E-cadherin [3]. E-cadherin is a cell adhesion molecule that is required for cell-cell contact, cell differentiation and maintenance of tissue homeostasis [4, 5]. The loss of e-cadherin results in the distinct diffuse, often subclinical growth pattern, which consequently makes ILC a challenging subtype to detect and follow-up by physical examination, and standard imaging modalities [6, 7]. Histologically, ILC can be divided into many subtypes; of which classic ILC and pleomorphic (high-grade) ILC are most prominent. In contrast to pleomorphic ILC, classic ILC cells have a low- to intermediate- nuclear-grade morphology and low mitotic count [8] and a low proliferative rate (Ki-67 labelling). The majority of classic ILC (80–95%) are hormone-receptor positive/human epidermal growth-factor receptor 2 negative (HR+/HER2-) [9, 10], while pleomorphic ILC is more often HR- or HER2+.

Although there have been many recent efforts to further characterize ILC resulting in more understanding of underlying biology and therapeutic implications [11], young patients (under 40 years) with ILC remain heavily understudied. A recent retrospective series showed that the long-term disease-specific survival of any-stage HR+/HER2- pre-menopausal patient (<50 years) with ILC is better in the first 10 years compared to invasive breast cancer of no special type (BC-NST, formerly known as invasive ductal carcinoma). After 10 years however, there is a statistically significant decline in disease-specific survival compared to BC-NST [12] where late recurrences are also observed in older women with ILC [13, 14]. For ILC patients < 40 years, the epidemiology, prognosis as well as prognostic and predictive biomarkers are still unknown. This may suggest that the current treatment paradigm for young patients with HR+/HER2- ILC might not be sufficient beyond 10 years. This is particularly important for young patients with a long life-expectancy.

Some established predictive and prognostic biomarkers in young patients with BC-NST have not been validated for ILC. Tumor grade, for instance, is used in the current ESMO guidelines for early breast cancer to tailor systemic treatment [15], despite tumor grading being impaired in ILC [16]. The non- cohesive growth pattern that characterizes ILC, results in the absence of glandular/tubular formation, one of the key components of tumor grade. (Neo) adjuvant chemotherapy efficacy remains unclear in ILC, due to studies showing limited efficacy of chemotherapy in ILC [17–20], but also studies showing benefit of a more intensified chemotherapy regimen in ILC including an anthracycline + taxane, over taxane only [21]. Established predictive and prognostic biomarkers are needed to navigate these disparities in young ILC patients with a potential high-risk of disease recurrence.

In this study, we set out to determine the 15-year prognosis and histopathological prognostic factors in young (<40 years) systemically untreated (no chemotherapy nor endocrine therapy) patients with HR+/HER2-, node-negative (N0) ILC versus matched BC-NST using a population-based cohort with minimal risk of confounding by indication, since in the era before 2000 node-negativity was considered a favorable marker and the Dutch guidelines recommended to refrain from adjuvant endocrine therapy and chemotherapy in this population. This study is unique in that it enables the study of pure prognosis and prognostic biomarkers in an underrepresented breast cancer population of young women with ILC. These results may be further used to tailor (neo) adjuvant systemic treatment choices in young patients with ILC.

## Methods

### Patients

For the current study, we selected all HR+/HER2- early breast cancer patients from PARADIGM, a retrospective cohort obtained through the prospectively collected Netherlands Cancer Registry (NCR). The PARADIGM initiative has been described previously [22]. In short, it is a population-based cohort that includes all patients < 40 years, diagnosed with node negative (N0) primary breast cancer between 1989 and 2000 in The Netherlands who were not treated with adjuvant systemic treatment. During this period, node-negativity was considered a favorable prognostic marker and guidelines recommended to forgo systemic therapy (both chemotherapy and/or endocrine therapy) in patients with N0 disease. All patients received primary surgery, including in most cases an axillary lymph node dissection and, if indicated, radiotherapy. From the same cohort, we selected the low-risk luminal A-like population as reference which is defined here as stage N0 with primary tumor < 5 cm, grade 1/2 and/or Ki-67 < 20%.

In addition to the previously received ethical approval for the PARADIGM initiative in 2012 (CFMPB173), we received consent for the current study on 21–02-2023 by the Institutional Review Board of the Netherlands Cancer Institute (IRBdm23-061). This research was conducted in accordance with the Declaration of Helsinki.

### Clinical follow-up data

Follow-up data on overall survival (OS) was collected via the municipality population register until 1–1-2018 and linked to the NCR. Distant- and regional recurrences were collected by dedicated NCR data managers directly from patient files, and updated until 1–4-2014.

### Histopathological biomarker assessment

Tissue blocks (formalin-fixed paraffin-embedded (FFPE)) were obtained through the nationwide network and registry of histo- and cytopathology in the Netherlands (PALGA) [23] after linking the NCR derived clinical data. The primary tumor material was revised by a team of breast cancer pathologists [22]. Histologic subtype and tumor grade were scored on digitalized whole hematoxylin & eosin (H&E) slides. Tissue Microarrays (TMAs) were constructed to assess the estrogen, progesterone and HER2 receptor status. A tumor was considered HR+ if estrogen receptor (ER) or progesterone receptor (PR) was ≥ 10%, and HER2 negative was defined (immunohistochemistry (IHC) score 0 or 1 or negative/equivocal in situ hybridization in case of IHC score of 2). Tumor grade was scored according to the modified Bloom and Richardson scoring system [24], derived from mitotic index (1=≤7, 2 = 8–12, 3 = ≥13 mitoses per 2 square mm), glandular tubular formation (1=<10%, 2 = 10–75% and 3 = >75% of the tumor area) and nuclear pleomorphism (1 = minimal, 2 = moderate, 3 = marked). Lymphovascular invasion (LVI) was either present or absent based on whether tumor cells were seen in either the lymphatic or vascular endothelial space of the tumor as described previously [25, 26]. ILC was defined according to the 4th edition (2012) World Health Organization (WHO) definition: “dispersed or linear dyscohesive cells with low-to-intermediate nuclear grade morphology and a low mitotic count” [27]. In addition, another expert pathologist re-assessed all H&E slides digitally according to the updated ILC histopathological assessment which included e-cadherin and p120- staining if indicated [28]. All slides were scanned and uploaded on Slidescore (http://www.slidescore.com).

### Endpoints

The endpoints used in this study were defined according to the STEEP 2.0 criteria [29]. For OS all deaths including from breast cancer, non-breast cancer or death from an unknown cause were considered as an event. For distant recurrence-free survival (DRFS), both death from any (unknown) cause and distant recurrence were considered an event. For recurrence-free survival (RFS) death from any (unknown) cause, distant recurrence, local-regional invasive recurrence and invasive ipsilateral breast tumor recurrence were considered independent events. If a patient did not experience an event, they were censored at last contact.

### Statistical analysis

Descriptive statistics were used to display baseline characteristics of the cohorts. Wilcoxon rank sum test, Chi-square test and Fisher’s exact tests were used to test the difference between two continuous or categorical variables. Uni- and Multivariable Cox regression models were used to determine hazard ratios (HR) with confidence intervals (CI) for ILC vs. BC-NST and other potential clinicopathological prognostic biomarkers within ILC or BC-NST. *p*-values of 0.05 were considered significant. Survival curves were visualized and survival estimates at specific time points were calculated using the Kaplan-Meier method. The proportionality of hazard assumptions was tested according to the Schoenfeld residuals. Log-rank *p*-values of <0.05 were considered significant.

## Results

### Patient selection and baseline characteristics

We selected all patients with early HR+/HER2-, N0 breast cancer (*n* = 1138, supplementary Figure 1) from the PARADIGM series who were all under 40 years of age and systemically untreated. For the current study, we used the central morphological assessment of ILC according to the WHO guidelines. Specifically, as the Cohen’s kappa showed moderate agreement (kappa = 0.52, supplementary Table 1) between WHO definition of ILC and preferred updated recommended histopathologic ILC diagnosis [28], and because it reflects the current situation in the clinic. Similar trends were found for the updated recommended histopathologic ILC cases and for the ILC cases according to the WHO definition (data not shown). Patients with histologic subtype BC-NST (*n* = 805) or classical ILC (*n* = 95) were included in the current study (supplementary Figure 1), *n* = 238 were classified as breast cancer subtype other. Baseline characteristics are set out in Table 1. Median age was 36 in the whole cohort (range 20–39). Patients with ILC more often had a larger tumor compared to BC-NST (68% of patients in the ILC cohort had a T1 tumor versus 77% in the BC-NST cohort, *p* = 0.014). Tumor grade differed statistically between BC-NST and ILC cohorts, with almost all patients in the ILC cohort presenting with a grade 2 tumor (81% vs. 45%, *p* < 0.001). As expected, considering the non-cohesive growth pattern that characterizes ILC, glandular/tubular formation was absent in almost all ILC cases, and the distribution of scores differed significantly from BC-NST (98% score 3 in ILC vs. 62% score 3 in BC-NST, *p* < 0.001). Patients with ILC more often presented with a multifocal tumor (21% vs. 9%, *p* < 0.001) and were more often treated with a mastectomy (48% vs. 32% *p* = 0.003) compared to BC-NST.

**Table 1:** Baseline characteristics per histologic subtype

		ILC n = 95 (%)	BC-NST n = 805 (%)	Total n = 900 (%)	p-value
Age	Median [min-max]	37 [25–39]	36 [20–39]	36 [20–39]	0.2
T-stage	T1	65 (68)	620 (77)	685 (76)	0.014
	T2	27 (28)	177 (22)	204 (23)	
	T3	3 (3)	4 (1)	7 (1)	
	Missing	0	4 (1)	4 (0)	
Tumor grade	1	11 (12)	205 (26)	216 (24)	<0.001
	2	77 (81)	363 (45)	440 (49)	
	3	7 (7)	236 (29)	243 (27)	
	Missing	0	1 (0)	1 (0)	
Nuclear pleomorphism	1	11 (12)	37 (5)	48 (5)	0.001
	2	67 (71)	515 (64)	582 (65)	
	3	17 (18)	251 (31)	268 (30)	
	Missing	0	2 (0)	2 ()	
Mitotic index	1	83 (87)	416 (52)	499 (55)	<0.001
	2	10 (11)	210 (26)	220 (24)	
	3	2 (2)	177 (22)	179 (20)	
	Missing	0	2 (0)	2 (0)	
Glandular/tubular formation	1	0	84 (10)	84 (9)	<0.001
	2	2 (2)	218 (27)	220 (24)	
	3	93 (98)	501 (62)	594 (66)	
	Missing	0	1 (0)	2 (0)	
Lymphovascular invasion	Absent	84 (88)	649 (81)	733 (81)	0.067
	Present	11 (12)	155 (19)	166 (18)	
	Missing	0	1 (0)	1 (0)	
ER score	<90	25 (26)	235 (29)	260 (29)	0.6
	≥90	70 (74)	570 (71)	640 (71)	
Multifocality	No	68 (72)	700 (87)	768 (85)	<0.001
	Yes	20 (21)	75 (9)	95 (11)	
	Missing	7 (7)	30 (4)	37 (4)	
Surgery^a^	BCS	47 (50)	536 (67)	583 (65)	0.003
	Mastectomy	46 (48)	256 (32)	302 (34)	
	Surgery NOS	2 (2)	13 (2)	15 (2)	

a) In the ILC cohort, 46/47 (98%) patients treated with BCS received radiotherapy, and 8/46 (17%) patients treated with mastectomy received radiotherapy. In the BC-NST cohort, 527/536 (98%) patientswith BCS received radiotherapy, and 32/256 (13%) patients that underwent a mastectomy received radiotherapy

ILC, invasive lobular cancer; BC-NST, invasive breast cancer of nospecial type; ER, Estrogen Receptor; BCS, breast conserving surgery; SurgeryNOS, surgery not otherwise specified

### Tumor grade is not prognostic in young patients with N0 ILC

With a median follow-up of 14.9 years, the following clinicopathological factors were significantly associated with DRFS in early BC-NST: age (<35 vs. ≥35; adjusted HR 1.33, 95% CI 1.01–1.75), T-stage (adjusted HR 1.39, 95% CI 1.03–1.86), grade (adjusted HR 2.96, 95% CI 1.92–4.56) and LVI (adjusted HR 2.18, 95% CI 1.63–2.91) (Table 2). In patients with ILC, only the presence of LVI (adjusted hazard ratio 2.81, 95%CI 1.14–6.94), and a multifocal tumor (adjusted HR 3.28, 95% CI 1.49–7.22) were significantly associated with a worse DRFS in multivariable analysis (Table 2). Tumor grade was not associated with DRFS in ILC, which is not surprising due to the absence of glandular/tubular formation and impact on tumor grading. Similarly, none of the individual components defining grade (including nuclear pleomorphism and mitotic index) were associated with DRFS. T-stage was not significantly associated with DRFS in ILC (HR 1.67, 95% CI 0.81–3.43), however, as similar effect estimates were seen as in BC-NST, this might be due to the limited power in this subgroup analysis. For RFS and OS both tumor grade and LVI remained a significant factor contributing to prognosis in BC-NST (supplementary Tables 2 and 3). In ILC, LVI was significantly associated with worse RFS (adjusted HR 3.39, 95% CI 1.60–7.20, *p* < 0.01, supplementary Table 2). Multifocality was significantly associated with worse OS in ILC (adjusted HR 2.30, 95% CI 1.12–4.73, *p* < 0.05, supplementary Table 3), but not in BC-NST.

**Table 2:** Uni/Multivariable cox regression DRFS ILC vs. BC-NST

		ILC			BC-NST		
		Univariable - DRFS		Multivariable^a^	Univariable -DRFS		**Multivariable**^b^
		Number of patients/events	HR for DRFS (95% CI)	Adjusted HR for DRFS (95% CI)	Number of patients/events	HR for DRFS (95% CI)	Adjusted HR for DRFS (95% CI)
Variable							
Age	35 and above	67/21	Ref		544/133	Ref	Ref
	Below 35	28/9	1.13 (0.52–2.48)		261/86	1.43 (1.09–1.88)**	1.33 (1.01–1.75)*
T stage	T1	65/17	Ref		620/153	Ref	Ref
	T2+	30/13	1.67 (0.81–3.43)		181/65	1.63 (1.22–2.18)***	1.39 (1.03–1.86)*
Tumor grade	1	11/3	Ref		205/28	Ref	Ref
	2	77/25	1.40 (0.42–4.63)		363/94	2.02 (1.32–3.08)**	1.70 (1.11–2.62)*
	3	7/2	1.19 (0.20–7.10)		236/97	3.81 (2.50–5.80)***	2.96 (1.92–4.56)***
Nuclear pleomorphism	1	11/3	0.86 (0.25–2.90)		37/5	0.59 (0.24–1.44)	0.97 (0.38–2.48)
	2	67/19	Ref		515/120	Ref	Ref
	3	17/8	2.10 (0.92–4.81)		251/94	1.84 (1.40–2.41)***	1.07 (0.76–1.52)
Mitotic index	1	83/25	Ref		416/77	Ref	Ref
	2 + 3	12/5	1.39 (0.53–3.63)		387,142	2.38 (1.80–3.14)***	1.30 (0.87–1.94)
Glandular/tubular formation	1	0	NA		84/14	0.47 (0.27–0.81)**	1.32 (0.63–2.74)
	2	2/2	Numbers too low		218/44	0.55 (0.39–0.77)***	1.03 (0.69–1.53)
	3	93/28			501/161	Ref	Ref
Lymphovascular invasion	Absent Present	84/22 11/8	Ref 4.60 (2.02–10.47)***	Ref 2.81 (1.14–6.94)*	649/150 155/69	Ref 2.45 (1.84–3.27)***	Ref 2.18 (1.63–2.91)***
ER	<90%	25/9	Ref		235/76	Ref	
	≥90%	70/21	0.77 (0.35–1.69)		570/143	0.76 (0.57–1.00)	
Surgery	BCS	47/10	Ref		536/133	Ref	
	Mastectomy	46/20	2.38 (1.11–5.09)*		256/80	1.31 (1.00–1.73)	
Multifocality	No	68/16	Ref	Ref	700/179	Ref	
	Yes	20/12	3.95 (1.85–8.41)***	3.28 (1.49–7.22)**	75/22	1.42 (0.91–2.20)	

*p-value < 0.05

**p-value < 0.01

***p-value < 0.001

a Including multifocality and lymphovascular invasion

b Including age, T-stage, Grade and lymphovascular invasion

ILC, invasive lobular cancer; BC-NST, invasive breast cancer of nospecial type; DRFS, distant recurrence-free survival; HR, hazard ratio; 95%CI, 95% confidence interval; ER, estrogen receptor; BCS, breast conserving surgery

### Long-term prognosis in young patients with N0 ILC vs. BC-NST

**Fig. 1:**
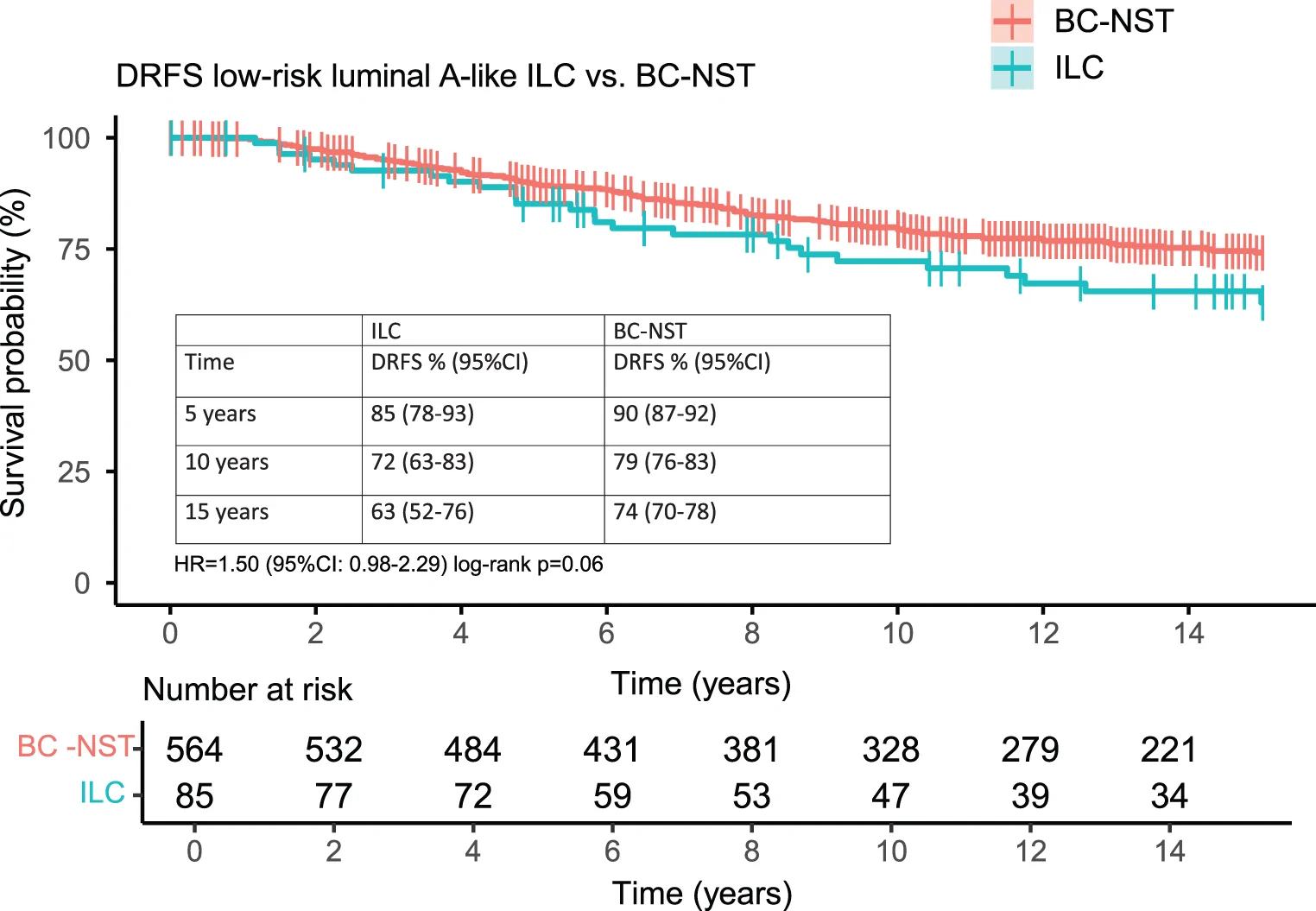
DRFS low risk luminal A-like ILC vs. BC-NST. Kaplan-meier curve displaying distant-recurrence free survival (DRFS) in low-risk luminal A-like invasive lobular cancer (ILC) versus matched invasive breast cancer of no special type (BC-NST). HR, hazard ratio; 95% CI, 95% confidence interval

The 15-year DRFS, RFS and OS did not differ significantly between all ILC vs. BC-NST (supplementary Figure 2). However, considering the imbalance between ILC and BC-NST regarding tumor grade this might not be a fair comparison. We proceeded by selecting all patients with T1-2 luminal A-like ILC (*n* = 85) or NST (*n* = 564), specifically excluding the high-risk tumors (T3 tumors and grade 3 tumors *n* = 251, supplementary Figure 1). Baseline characteristics are set out in supplementary Table 4. The absolute DRFS at 15 years in luminal A-like ILC was 63% (95% CI 52%-76%) vs. 74% (95% CI 70%-78%) in luminal A-like BC-NST (HR 1.50, 95%CI 0.98–2.29, *p* = 0.06, Fig. 1). Absolute RFS rates at 15 years were 48% (95%CI 37%-61%) for ILC vs. 59% (95%CI 55%-64%) for BC-NST (HR 1.35, 95%CI 0.96–1.92, *p* = 0.09, supplementary Figure 3a). The absolute OS rate at 15 years was 71% (95%CI 62%-82%) for ILC vs. 80% (95%CI 77%-84%) for BC-NST (HR1.40, 95%CI 0.95–2.05, *p* = 0.09, supplementary Figure 3b).

### Nuclear pleomorphism, LVI and multifocality are prognostic in low-risk luminal A-like ILC

Most ILCs are grade 2 due to the inability of the tumor to form gland-like structures. This may explain why tumor grade does not contribute to prognosis in N0 ILC. To understand whether the separate components defining grade are prognostic, we analyzed nuclear pleomorphism and mitotic index separately. Strikingly, in the low-risk luminal A-like ILC’s, nuclear pleomorphism was significantly associated with DRFS (adjusted HR 3.02, 95%CI 1.08–8.41, *p* < 0.05, supplementary Table 5) together with LVI (adjusted HR 3.12, 95%CI 1.20–8.58, *p* < 0.05) and multifocality (adjusted hazard ratio 2.87, 95%CI 1.21–6.77, *p* < 0.05). Nuclear pleomorphism remained significantly associated with RFS (adjusted HR 2.93, 95%CI 1.18–7.26, *p* < 0.05, supplementary Table 6) in a multivariable model together with LVI (HR 4.64, 95%CI 2.18–9.91, *p* < 0.001). LVI was the only marker significantly associated with OS in low-risk luminal A-like ILC (HR 2.75, 95%CI 1.13–6.72, *p* < 0.05, supplementary Table 7). In this cohort only very few patients with low-risk ILC had a mitotic index score of 2 or 3, which limited the ability to investigate mitotic index score as a potential prognostic biomarker (Supplementary Tables 5, 6, 7).

## Discussion

In this study we set out to determine the prognosis and prognostic factors of young (<40 years) women with HR+/HER2- ILC. This study is unique in that it utilized a high quality prospectively collected cancer registry of untreated N0 patients, under 40 years of age with 15 years of follow-up. We show that tumor grade was not significantly prognostic in young women with ILC, while LVI and multifocality were significant prognostic markers in this patient population. Furthermore, we observed a trend for a worse 15-year survival in low-risk luminal A-like ILC compared to matched BC-NST.

Previous studies have shown that standard prognostic factors such as T-stage, N-stage and high tumor grade, but also gene-expression based prediction models may be useful in distinguishing high- risk from low-risk ILC [30, 31]. However, tumor grading is problematic in ILC, due to the absence of glandular/tubular formation, one of the three components of tumor grade. We show that tumor grade is not significantly prognostic in young woman with N0 ILC, which is particularly important in this young patient population as tumor grade is currently used in many guidelines to tailor adjuvant hormonal and chemotherapy choice in early breast cancer [15, 32]. Recent research has indicated that the 21-gene recurrence score and MammaPrint are prognostic in ILC [31, 33] which might provide further insight into patient stratification in young patients with N0 ILC. Our results furthermore underpin the important effect of LVI and multifocality on prognosis in young women with N0 ILC. Recently it has been reported that LVI is an independent prognostic marker in young patients with triple-negative breast cancer [34], although at this point interobserver variability between pathologists is challenging and there is need for optimization of the scoring criteria [35]. We also show that nuclear pleomorphism might differentiate patients based on prognosis in low-risk luminal A-like young patients with ILC. However, we acknowledge that the limited numbers in this study require further investigation of these markers in larger validation studies.

Despite the biological differences between ILC and BC-NST, a clear rationale for differential systemic treatment approach is absent. As a result, histologic subtype is not usually taken into account when initiating systemic treatment in breast cancer [15]. Most N0 patients with ILC are low-risk luminal A-like, and will have an indication for adjuvant endocrine therapy only [36]. Our results suggest that the long-term outcome of systemically untreated patients diagnosed under < 40 years with low-risk luminal A-like N0 ILC is worse than matched low-risk luminal A-like BC-NST. Although we do not have a direct comparison of young N0 women with ILC treated with hormonal therapy only, the question arises whether hormonal therapy alone in these patients is enough. The observation that ILC recurs late, and that after 10 years survival estimates are worse than for matched BC-NST has been shown previously in women above 40 years as well [14]. One of the challenges in ILC, is the autocrine activation of growth-factor receptor pathways that are key players in cell dormancy [37], which may in part explain late recurrences, endocrine resistance and metastatic outgrowth in ILC [38]. A focus therefore on optimizing current adjuvant endocrine therapy strategies with or without the addition of chemo, targeted or immune-therapies may prove beneficial in these patients.

In early breast cancer, CDK4/6 inhibition (CDK4/6i) with endocrine therapy is approved for high-risk HR+/HER2-breast cancer including high-risk ILC [39]. The PELOPS study (NCT02764541) is investigating CDK4/6i + endocrine therapy in all ILC, of which most tumors are low-proliferative (Ki-67 of <20%) [40]. This is interesting as subgroup analysis of the landmark CDK4/6 trials suggests efficacy of CDK4/6i + endocrine therapy in low-proliferative breast cancers with a Ki-67 of <20% [41, 42]. ILC has a distinct molecular make-up in which mutations that activate the PI3K-pathway are frequently observed (mutations in PIK3CA (43.3%), AKT1 (4.1%), PTEN (3.9%)) next to e-cadherin impairment [4, 43–45]. An activated PI3K-pathway in ILC might also be a result of hyperactivation of growth factor receptors an autocrine feedback loop through E-cadherin loss [38, 46]. This may indicate particular sensitivity towards PI3K-AKT directed therapies [38]. In metastatic HR+/HER2- breast cancer, AKT inhibition + fulvestrant has been approved for patients with AKT/PIK3CA/PTEN mutant tumors [47], and a trial investigating the triple combination of CDK4/6 inhibition, AKT-inhibition and fulvestrant is ongoing [48]. Based on previous research it is likely that these combination therapies will be particularly efficacious in all ILC, and warrant further investigation particularly in the (young) ILC population with a possible high-risk of recurrence. However, as these therapies come with considerable toxicity, their initiation must be carefully considered based on the individual patient. Another interesting observation is the increased benefit of an adjuvant aromatase inhibitor over tamoxifen when combined with ovarian function suppression in premenopausal patients with ILC [49], which is usually reserved for the more high-risk breast cancer population [15].

Besides the limitations associated with the retrospective collection of disease-specific follow-up in this study, we must also highlight the high quality clinical and OS follow-up data that were prospectively collected by the NCR. In addition, we acknowledge the small number of ILC patients in this cohort which warrant validation of our results in larger cohorts. Another important factor that should be taken into consideration when interpreting our results is that approximately 11% of young patients with ILC will harbor a pathogenic germline mutation in a breast cancer susceptibility gene such as CDH1 and BRCA2 [50]. Unfortunately, because germline mutational status was not available in this patient cohort, we could not assess how carrier status may have affected prognosis in this young patient population It is also relevant to note that the ILC-specific growth pattern extends to tumor cells that may be present in lymph nodes [51]. Axillary imaging for ILC might therefore be less accurate [52]. As in this cohort almost all patients received an axillary lymph node dissection, which was standard practice at the time, the chance of any wrongfully included node-positive patients in this cohort is likely small.

In conclusion, this study provides important data on 15-years prognosis and prognostic factors in young (<40 years) women with N0 ILC that were not treated with systemic therapy. We show that prognosis was worse in luminal A-like ILC compared to matched luminal A-like BC-NST. Moreover, we show that grade did not contribute to prognosis in young patients with ILC, but LVI and multifocality do. Given the poor long-term prognosis observed for some young patients with ILC, identification of treatment strategies that are effective for this subtype remains an important unmet clinical need.

## Electronic supplementary material

Below is the link to the electronic supplementary material.

Supplementary Material 1

## Data Availability

Data are available from the corresponding author upon reasonable request and permission from The Netherlands Comprehensive Cancer Center (IKNL).
