# Supplementary material for: Invasive lobular breast cancer in young patients without adjuvant systemic treatment: 15-year survival outcomes
Source: Breast Cancer Res Treat. 2026 Aug 6;218(3):31. doi: 10.1007/s10549-026-08024-1 (PMC13447550; doi:10.1007/s10549-026-08024-1)
Supplement: Supplementary file 1 [file 10549_2026_8024_MOESM1_ESM.pdf]

# Supplementary appendix

## Invasive lobular breast cancer in young, systemically untreated patients: 15-year survival outcomes

- Supplementary tables
- Supplementary figure legends
- Supplementary figures

## Supplementary table 1: concordance standard of care ILC diagnosis and recommended updated ILC diagnosis

| | | ILC according to recommended ILC histopathology | | | |
|---|---|---|---|---|---|
| | | ILC | BC-NST | Mixed ILC | Other |
| ILC according to current clinical practice | ILC | 41 | 43 | 1 | 2 |
| | BC-NST | 11 | 749 | 0 | 9 |
| | Mixed ILC | 4 | 58 | 0 | 0 |
| | Other | 0 | 125 | 0 | 45 |

Concordance between old and new diagnosis ILC and BC-NST in 844 observations is 0.54 (Cohens kappa), with a 93.6% raw agreement.

ILC, invasive lobular cancer; BC-NST, invasive breast cancer of no special type

## Supplementary table 2: Uni/multivariable cox regression RFS ILC vs. BC-NST

| | | ILC | | | BC-NST | | |
|---|---|---|---|---|---|---|---|
| | | **Univariable- RFS** | | **Multivariable[a]** | **Univariable -RFS** | | **Multivariable[b]** |
| | | Number of patients/events | HR for RFS (95% CI) | Adjusted HR for RFS (95% CI) | Number of patients/events | HR for RFS (95% CI) | Adjusted HR for RFS (95% CI) |
| Variable | | | | | | | |
| Age | 35 and above<br>Below 35 | 67/28<br>28/15 | Ref<br>1.36 (0.71-2.55) | | 544/199<br>261/115 | Ref<br>1.30 (1.03-1.63)* | Ref<br>1.21 (0.96-1.53) |
| T stage | T1<br>T2+ | 65/26<br>30/17 | Ref<br>1.58 (0.86-2.92) | | 620/228<br>181/84 | Ref<br>1.42 (1.11-1.83)** | Ref<br>1.28 (0.99-1.64) |
| Tumor grade | Grade 1<br>Grade 2<br>Grade 3 | 11/5<br>77/35<br>7/3 | Ref<br>1.17 (0.46-2.99)<br>0.99 (0.24-4.14) | | 205/53<br>363/150<br>236 /111 | Ref<br>1.72 (1.25-2.35)***<br>2.30 (1.65-3.18)*** | Ref<br>1.49 (1.08-2.05)*<br>1.89 (1.35-2.64)*** |
| Nuclear pleomorphism | 1<br>2<br>3 | 11/5<br>67/28<br>17/10 | 1.00 (0.38-2.58)<br>Ref<br>1.99 (0.97-4.12) | | 37/7<br>515/191<br>251/116 | 0.50 (0.24-1.07)<br>Ref<br>1.41 (1.12-1.78)** | 0.70 (0.32-1.52)<br>Ref<br>1.07 (0.79-1.44) |
| Mitotic index | 1<br>2+3 | 83/36<br>12/7 | Ref<br>1.24 (0.55-2.79) | | 416/137<br>387/177 | Ref<br>1.66 (1.33-2.08)*** | Ref<br>1.15 (0.84-1.59) |
| Glandular/tubular formation | 1<br>2<br>3 | 0<br>2/2<br>93/41 | NA<br>Numbers too low | | 84/22<br>218/79<br>501/213 | 0.57 (0.37-0.89)*<br>0.74 (0.57-0.96)*<br>Ref | 1.20 (0.67-1.53)<br>1.15 (0.84-1.58)<br>Ref |
| Lymphovascular invasion | Absent<br>Present | 84/32<br>11/11 | Ref<br>4.42 (2.21-8.85)*** | Ref<br>3.39 (1.60-7.20)** | 649/228<br>155/86 | Ref<br>2.09 (1.63-2.68)*** | Ref<br>1.93 (1.50-2.49)*** |
| ER | <90<br>≥ 90 | 25/10<br>70/33 | Ref<br>1.12 (0.55-2.28) | | 235/103<br>570 /211 | Ref<br>0.82 (0.65-1.04) | |

| | | | | | | | |
|---|---|---|---|---|---|---|---|
| Surgery[1] | BCS<br>Mastectomy | 47/20<br>46/23 | Ref<br>1.47 (0.81-2.67) | | 536/216<br>256/91 | Ref<br>0.90 (0.71-1.15) | |
| Multifocality | No<br>Yes | 68/29<br>20/12 | Ref<br>2.00 (1.01-3.93)* | Ref<br>1.57 (0.77-3.20) | 700/265<br>75/30 | Ref<br>1.20 (0.89-1.90) | |

* <0.05
** <0.01
***<0.001

[a] Including multifocality and lymphovascular invasion
[b] Including age, T-stage, Grade and lymphovascular invasion

[1] In the ILC cohort, 46 (98%) patients treated with BCS received radiotherapy, and 8 (17%) patients treated with mastectomy received radiotherapy. In the BC NST cohort, 527 (98%) patients with BCS received radiotherapy, and 32 (13%) patients that underwent a mastectomy received radiotherapy

ILC, invasive lobular cancer; BC-NST, invasive breast cancer of no special type; RFS, recurrence-free survival; HR, hazard ratio; 95%CI, 95% confidence interval; ER, estrogen receptor; BCS, breast conserving surgery;

## Supplementary table 3: Uni/multivariable cox regression OS ILC vs. BC-NST

| | | ILC | | | BC-NST | | |
|---|---|---|---|---|---|---|---|
| | | Univariable- OS | | Multivariable[a] | Univariable -OS | | Multivariable[b] |
| | | Number of patients/events | HR for OS (95% CI) | Adjusted HR for OS (95% CI) | Number of patients/events | HR for OS (95% CI) | Adjusted HR for OS (95% CI) |
| Variable | | | | | | | |
| Age | 35 and above<br>Below 35 | 67/25<br>28/11 | Ref<br>1.11 (0.55-2.26) | | 544/179<br>261/100 | Ref<br>1.21 (0.95-1.54) | |
| T stage | T1<br>T2+ | 65/21<br>30/15 | Ref<br>1.68 (0.86-3.25) | | 620/203<br>181/75 | Ref<br>1.39 (1.07-1.81)* | Ref<br>1.19 (0.91-1.56) |
| Tumor grade | Grade 1<br>Grade 2<br>Grade 3 | 11/4<br>77/30<br>7/2 | Ref<br>1.17 (0.41-3.32)<br>0.78 (0.14-4.30) | | 205/40<br>363/120<br>236 /119 | Ref<br>1.86 (1.30-2.66)***<br>3.37 (2.35-4.82)*** | Ref<br>1.68 (1.17-2.42)**<br>2.81 (1.94-4.07)*** |
| Nuclear pleomorphism | 1<br>2<br>3 | 11/4<br>67/24<br>17/8 | 0.97 (0.34-2.79)<br>Ref<br>1.55 (0.69-3.45) | | 37/9<br>515/155<br>251/115 | 0.77 (0.39-1.51)<br>Ref<br>1.73 (1.36-2.20)*** | 1.22 (0.60-2.50)<br>Ref<br>1.07 (0.79-1.44) |
| Mitotic index | 1<br>2+3 | 83/32<br>12/4 | Ref<br>0.88 (0.31-2.5) | | 416/103<br>387/176 | Ref<br>2.20 (1.72-2.80)*** | Ref<br>1.27 (0.89-1.81) |
| Glandular/tubular formation | 1<br>2<br>3 | 0<br>2/2<br>93/34 | NA<br>Numbers too low | | 84/18<br>218/60<br>501/201 | 0.48 (0.30-0.78)**<br>0.60 (0.45-0.80)***<br>Ref | 1.22 (0.64-2.32)<br>1.06 (0.74-1.50)<br>Ref |
| Lymphovascular invasion | Absent<br>Present | 84/29<br>11/7 | Ref<br>2.41 (1.06-5.52)* | Ref<br>1.66 (0.67-4.07) | 649/196<br>155/83 | Ref<br>2.18 (1.69-2.82)*** | Ref<br>1.91 (1.47-2.49)*** |

| | | | | | | | |
|---|---|---|---|---|---|---|---|
| ER | <90<br>≥ 90 | 25/9<br>70/27 | Ref<br>1.01 (0.47-2.14) | | 235/95<br>570 /184 | Ref<br>0.73 (0.57-0.93)* | Ref<br>0.83 (0.64-1.08 |
| Surgery[1] | BCS<br>Mastectomy | 47/15<br>46/21 | Ref<br>1.61 (0.83-3.13) | | 536/182<br>256/90 | Ref<br>1.06 (0.82-1.36) | |
| Multifocality | No<br>Yes | 68/22<br>20/12 | Ref<br>2.45 (1.21-4.97)* | Ref<br>2.30 (1.12-4.73)* | 700/228<br>75/31 | Ref<br>1.40 (0.96-2.04) | |

* <0.05
** <0.01
***<0.001

[a] Including multifocality and lymphovascular invasion
[b] Including age, T-stage, Grade and lymphovascular invasion

[1] In the ILC cohort, 46 (98%) patients treated with BCS received radiotherapy, and 8 (17%) patients treated with mastectomy received radiotherapy. In the BC NST cohort, 527 (98%) patients with BCS received radiotherapy, and 32 (13%) patients that underwent a mastectomy received radiotherapy

ILC, invasive lobular cancer; BC-NST, invasive breast cancer of no special type; OS, overall survival; HR, hazard ratio; 95%CI, 95% confidence interval; ER, estrogen receptor; BCS, breast conserving surgery;

## Supplementary table 4: baseline characteristics luminal A-like HR+/HER2- breast cancer

| | | ILC n=85 (%) | BC-NST n=564 (%) | Total n=649 (%) |
|---|---|---|---|---|
| Age | Median [min-max] | 37 [25-39] | 37 [23-39] | 37 [23-39] |
| T-stage | T1 | 63 (74) | 462 (82) | 525 (81) |
| | T2 | 22 (26) | 102 (18) | 124 (19) |
| Tumor grade | Grade 1 | 10 (12) | 204 (36) | 214 (33) |
| | Grade 2 | 75 (88) | 360 (64) | 435 (67) |
| Nuclear pleomorphism | 1 | 10 (12) | 37 (7) | 47 (7) |
| | 2 | 66 (78) | 449 (80) | 515 (80) |
| | 3 | 9 (11) | 77 (14) | 86 (13) |
| | Missing | 0 | 1 | 1 |
| Mitotic index | 1 | 80 (94) | 413 (73) | 493 (76) |
| | 2 | 5 (6) | 130 (23) | 135 (21) |
| | 3 | 0 | 21 (4) | 21 (3) |
| | Missing | 0 | 0 | 0 |
| Lymphovascular invasion | Absent | 76 (89) | 471 (84) | 547 (84) |
| | Present | 9 (11) | 92 (16) | 101 (16) |
| | Missing | 0 | 1 | 1 |
| ER score | <90 | 22 (26) | 133 (24) | 155 (24) |
| | ≥90 | 63 (74) | 431 (76) | 494 (76) |
| Multifocality | No | 61 (77) | 492 (90) | 553 (88) |
| | Yes | 18 (23) | 56 (10) | 74 (12) |
| | Missing | 6 | 16 | 22 |
| Surgery[1] | BCS | 43 (51) | 384 (68) | 427 (66) |
| | Mastectomy | 40 (47) | 174 (31) | 214 (33) |
| | Surgery NOS | 2 (2) | 6 (1) | 8 (1) |

[1] In the ILC group, 42 patients treated with BCS had radiotherapy, and 5 patients treated with a mastectomy received radiotherapy. In the BC NST group, 377 patients treated with BCS received radiotherapy, and 19 patients treated with a mastectomy received radiotherapy.

ILC, invasive lobular carcinoma; BC NST, breast cancer of no special type; ER, estrogen receptor; BCS, breast-conserving surgery; Surgery NOS, surgery not otherwise specified.

## Supplementary table 5: Univariable analysis DRFS low-risk luminal A-like ILC and BC-NST

| | | ILC | | | BC-NST | | |
|---|---|---|---|---|---|---|---|
| | | Univariable- DRFS | | Multivariable [a] | Univariable- DRFS | | Multivariable [b] |
| | | Number of patients/events | HR for DRFS (95% CI) | Adjusted HR for DRFS (95% CI) | Number of patients/events | HR for DRFS (95% CI) | Adjusted HR for DRFS (95% CI) |
| Variable | | | | | | | |
| Age | 35 and above<br>Below 35 | 60/18<br>25/8 | Ref<br>1.19 (0.52-2.74) | | 399/79<br>165/42 | Ref<br>1.34 (0.92 – 1.95) | |
| T stage | T1<br>T2 | 63/16<br>32/10 | Ref<br>1.87 (0.85 – 4.11) | | 462/93<br>102/28 | Ref<br>1.45 (0.95- 2.22) | |
| Tumor grade | Grade 1<br>Grade 2 | 10/3<br>75/23 | Ref<br>1.21 (0.36-4.04) | | 204/27<br>360/94 | Ref<br>2.12 (1.38 – 3.25)*** | Ref<br>1.85 (1.20-2.86)** |
| Nuclear pleomorphism | 1<br>2<br>3 | 10/3<br>66/18<br>9/5 | 0.96 (0.28-3.26)<br>Ref<br>2.87 (1.06-7.73)* | Not significant<br>Ref<br>3.02 (1.08-8.41)* | 37/5<br>449/93<br>77/23 | 0.68 (0.28-1.67)<br>Ref<br>1.60 (1.01-2.53)* | Not significant<br>Ref<br>Not significant |
| Mitotic index | 1<br>2<br>3 | 80/23<br>5/3 | Ref<br>2.16 (0.65 – 7.12)<br>- | | 413 (76)<br>130 (33)<br>21 (12) | Ref<br>1.51 (1.00-2.27)*<br>4.85 (2.63-8.95)** | Ref<br>Not significant<br>3.28 (1.73-6.22)*** |
| Lymphovascular invasion | Absent<br>Present | 76/19<br>9/7 | Ref<br>5.27 (2.19-12.69)*** | Ref<br>3.12 (1.20-8.58)* | 471/84<br>92/37 | Ref<br>2.80 (1.90 - 4.12)*** | Ref<br>2.51 (1.69-3.72)*** |
| ER | <90<br>≥ 90 | 22/8<br>63/18 | Ref<br>0.74 (0.32 – 1.70) | | 133/30<br>431/91 | Ref<br>0.96 (0.64 – 1.45) | |
| Surgery[1] | BCS<br>Mastectomy | 43/9<br>40/17 | Ref<br>2.42 (1.08-5.43)* | Ref<br>1.24 (0.42-3.64) | 384/74<br>174/45 | Ref<br>1.36 (0.94-1.97) | |

| | | | | | | | |
|---|---|---|---|---|---|---|---|
| Multifocality | No<br>Yes | 61/14<br>18/10 | Ref<br>3.59 (1.58-8.13)** | Ref<br>2.87 (1.21-6.77)* | 492/99<br>56/13 | Ref<br>1.45 (0.81 – 2.58) | |

* <0.05
** <0.01
***<0.001

[1] In the ILC cohort, 42 patients treated with BCS received radiotherapy, and 5 patients treated with mastectomy received radiotherapy. In the BC-NST cohort, 377 patients treated with BCS received radiotherapy, and 19 patients treated with mastectomy received radiotherapy.

[a] Including multifocality and lymphovascular invasion
[b] Including grade and lymphovascular invasion

ILC, invasive lobular cancer; BC-NST, invasive breast cancer of no special type; DRFS, distant recurrence-free survival; HR, hazard ratio; 95%CI, 95% confidence interval; ER, estrogen receptor; BCS, breast conserving surgery;

## Supplementary table 6: Univariable analysis RFS luminal A-like ILC and BC-NST

| | | ILC | | | BC-NST | | |
|---|---|---|---|---|---|---|---|
| | | Univariable- RFS | | Multivariable [a] | Univariable -RFS | | Multivariable [b] |
| | | Number of patients/events | HR for RFS (95% CI) | Adjusted HR for RFS (95% CI) | Number of patients/events | HR for RFS (95% CI) | Adjusted HR for RFS (95% CI) |
| Variable | | | | | | | |
| Age | 35 and above<br>Below 35 | 60/25<br>25/13 | Ref<br>1.35 (0.69-2.65) | | 399/134<br>165/67 | Ref<br>1.27 (0.95 – 1.71) | |
| T stage | T1<br>T2 | 63/24<br>32/14 | Ref<br>1.94 (1-3.76)* | Ref<br>1.71 (0.88-3.55) | 462/157<br>102/44 | Ref<br>1.37 (0.98 – 1.91) | |
| Tumor grade | Grade 1<br>Grade 2 | 10/5<br>75/33 | Ref<br>1.01 (0.39-2.59) | | 204/52<br>360/149 | Ref<br>1.76 (1.28 – 2.41)*** | Ref<br>1.60 (1.16-2.20)** |
| Nuclear pleomorphism | 1<br>2<br>3 | 10/5<br>66/27<br>9/6 | 1.13 (0.44-2.94)<br>Ref<br>3.25 (1.33-7.92)** | Not significant<br>Ref<br>2.93 (1.18-7.26)* | 37/7<br>449/158<br>77/36 | 0.55 (0.26-1.17)<br>Ref<br>1.48 (1.03-2.12)* | Not significant<br>Ref<br>Not significant |
| Mitotic index | 1<br>2<br>3 | 80/34<br>5/4<br>0 | Ref<br>1.79 (0.64-5.06)<br>- | | 413/135<br>130/52<br>21/14 | Ref<br>1.34 (0.97-1.85)<br>3.32 (1.91-5.77)*** | Ref<br>Not significant<br>2.48 (1.40-4.39)** |
| Lymphovascular invasion | Absent<br>Present | 76/29<br>9/9 | Ref<br>4.64 (2.18-9.91)*** | Ref<br>4.64 (2.18-9.91)*** | 471/152<br>92/49 | Ref<br>2.10 (1.52-2.89)*** | Ref<br>1.91 (1.37-2.65)*** |
| ER | <90<br>≥ 90 | 22/8<br>63/30 | Ref<br>1.24 (0.57-2.71) | | 133/53<br>431/148 | Ref<br>0.87 (0.64 – 1.19) | |
| Surgery [1] | BCS | 43/18 | Ref | | 384/143 | Ref | |

| | | | | | | | |
|---|---|---|---|---|---|---|---|
| | Mastectomy | 40/20 | 1.50 (0.79 – 2.84) | | 174/55 | 0.84 (0.62 – 1.15) | |
| Multifocality | No<br>Yes | 61/26<br>18/10 | Ref<br>1.77 (0.85-3.68) | | 492/171<br>56/20 | Ref<br>1.24 (0.78 – 1.97) | |

* <0.05
** <0.01
***<0.001

[1] In the ILC cohort, 42 patients treated with BCS received radiotherapy, and 5 patients treated with mastectomy received radiotherapy. In the BC-NST cohort, 377 patients treated with BCS received radiotherapy, and 19 patients treated with mastectomy received radiotherapy.

[a] Including lymphovascular invasion
[b] Including grade, lymphovascular invasion

ILC, invasive lobular cancer; BC-NST, invasive breast cancer of no special type; RFS, recurrence-free survival; HR, hazard ratio; 95%CI, 95% confidence interval; ER, estrogen receptor; BCS, breast conserving surgery;

Supplementary table 7: Univariable analysis OS luminal A-like ILC and BC-NST

| | | ILC | | | BC-NST | | |
|---|---|---|---|---|---|---|---|
| | | Univariabel- OS | | Multivariable | Univariabel-OS | | Multivariable [b] |
| | | Number of patients/events | HR for OS (95% CI) | Adjusted HR for OS (95% CI) | Number of patients/ events | HR for OS (95% CI) | Adjusted HR for OS (95% CI) |
| Variable | | | | | | | |
| Age | 35 and above<br>Below 35 | 60/21<br>25/10 | Ref<br>1.21 (0.57-2.58) | | 399/108<br>165/51 | Ref<br>1.19 (0.85 – 1.66) | |
| T stage | T1<br>T2 | 63/20<br>32/11 | Ref<br>1.81 (0.86-3.79) | | 462/129<br>102/30 | Ref<br>1.07 (0.72 – 1.59) | |
| Tumor grade | Grade 1<br>Grade 2 | 10/3<br>75/28 | Ref<br>1.35 (0.41 – 4.45) | | 204/39<br>360/120 | Ref<br>1.93 (1.34 – 2.77)*** | Ref<br>1.75 (1.21-2.52)** |
| Nuclear pleomorphism | 1<br>2<br>3 | 10/3<br>66/23<br>9/5 | 0.81 (0.24-2.71)<br>Ref<br>2.07 (0.78-5.45) | | 37/9<br>449/121<br>77/29 | 0.88 (0.45-1.73)<br>Ref<br>1.45 (0.97-2.18) | |
| Mitotic index | 1<br>2<br>3 | 80/29<br>5/2<br>0 | Ref<br>1.31 (0.31 – 5.51)<br>- | | 413/102<br>130/44<br>21/13 | Ref<br>1.47 (1.03-2.09)*<br>3.80 (2.13-6.78)*** | Ref<br>Not significant<br>2.70 (1.48-4.93)** |
| Lymphovascular invasion | Absent<br>Present | 76/25<br>9/6 | Ref<br>2.75 (1.13-6.72)* | | 471/116<br>92/43 | Ref<br>2.30 (1.62 – 3.27)*** | Ref<br>2.09 (1.46-2.98)*** |
| ER | <90<br>≥ 90 | 22/8<br>63/23 | Ref<br>0.93 (0.41-2.07) | | 133/42<br>431/117 | Ref<br>0.80 (0.56 – 1.13) | |
| Surgery [1] | BCS<br>Mastectomy | 43/14<br>40/17 | Ref<br>1.46 (0.72-2.96) | | 384/104<br>174/52 | Ref<br>1.11 (0.80 – 1.55) | |

| | | | | | | | | |
|---|---|---|---|---|---|---|---|---|
| Multifocality | No<br>Yes | 61/19<br>18/10 | Ref<br>2.16 (1.00 – 4.64) | | 492/127<br>56/21 | Ref<br>1.56 (0.98–2.47) | | |
| * <0.05<br>** <0.01<br>***<0.001 | | | | | | | | |
| [1] In the ILC cohort, 42 patients treated with BCS received radiotherapy, and 5 patients treated with mastectomy received radiotherapy. In the BC-NST cohort, 377 patients treated with BCS received radiotherapy, and 19 patients treated with mastectomy received radiotherapy. | | | | | | | | |
| [b] Including grade and lymphovascular invasion | | | | | | | | |

ILC, invasive lobular cancer; BC-NST, invasive breast cancer of no special type; OS, overall survival; HR, hazard ratio; 95%CI, 95% confidence interval; ER, estrogen receptor; BCS, breast conserving surgery;

## Supplementary Figure captions

### Supplementary figure 1: consort diagram PARADIGM series

HR+, hormone receptor positive; HER2- human epidermal growth factor receptor-2; ILC, invasive lobular carcinoma; BC-NST, invasive breast cancer of no special type.

### Supplementary figure 2: DRFS, RFS and OS in all ILC vs. BC-NST

Kaplan-Meier curves of **a**) distant recurrence-free survival (DRFS), **b)** recurrence-free survival (RFS) and **c)** overall survival in all invasive lobular carcinoma (ILC) versus invasive breast cancer of no special type (BC-NST). HR=hazard ratio; 95%CI, 95% confidence interval.

### Supplementary figure 3: RFS and OS in low-risk luminal A-like ILC vs. BC-NST

**a;** Kaplan-Meier curve of recurrence-free survival (RFS) in low-risk luminal A-like invasive lobular carcinoma (ILC) versus matched Invasive breast cancer of no special type (BC-NST). HR=hazard ratio; 95%CI, 95% confidence interval. **b;** Kaplan-Meier curve of overall survival (OS) in low-risk luminal A-like ILC vs. matched BC-NST

**Supplementary figure 1**

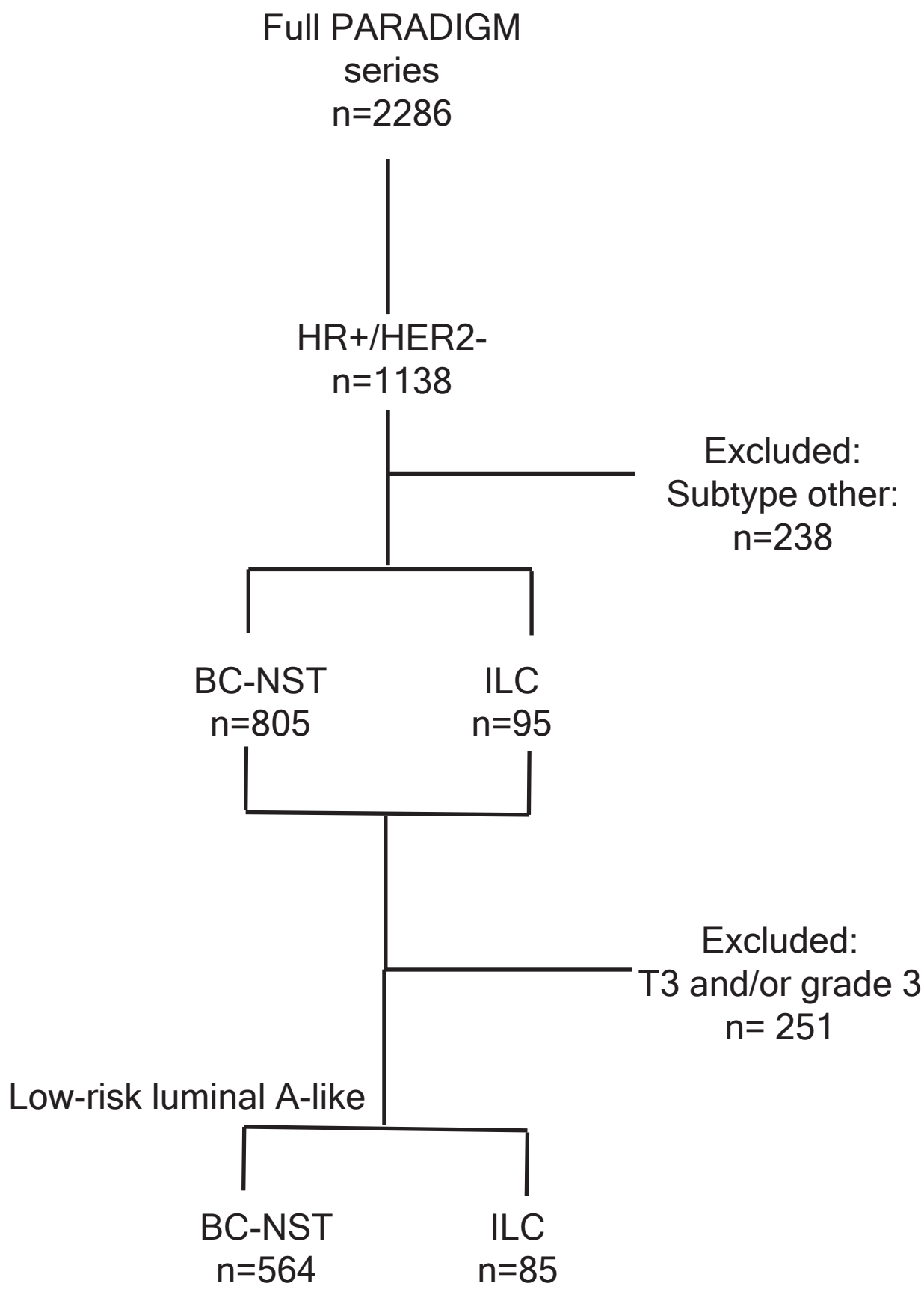

**Supplementary figure 2**

a

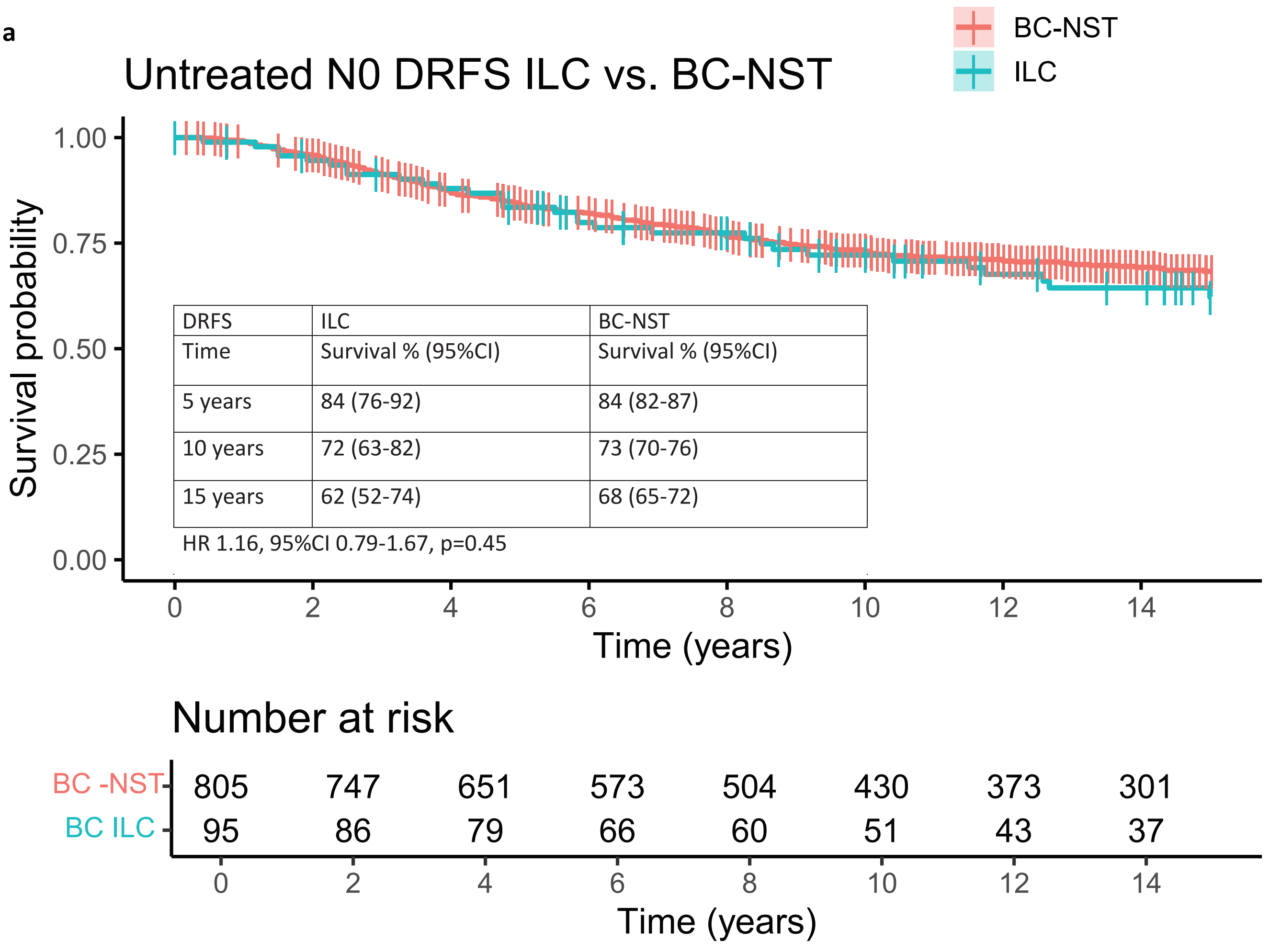


b

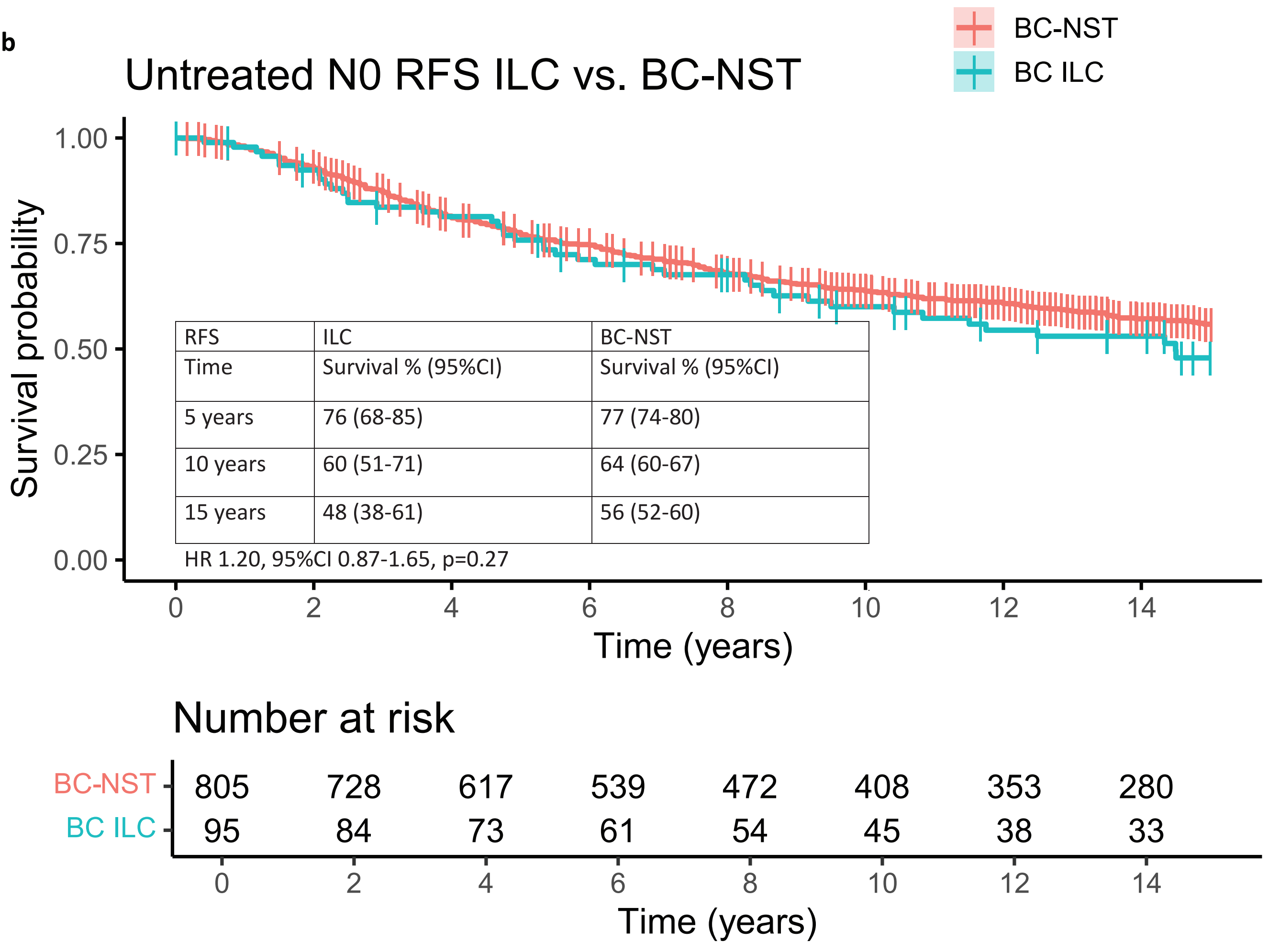


c

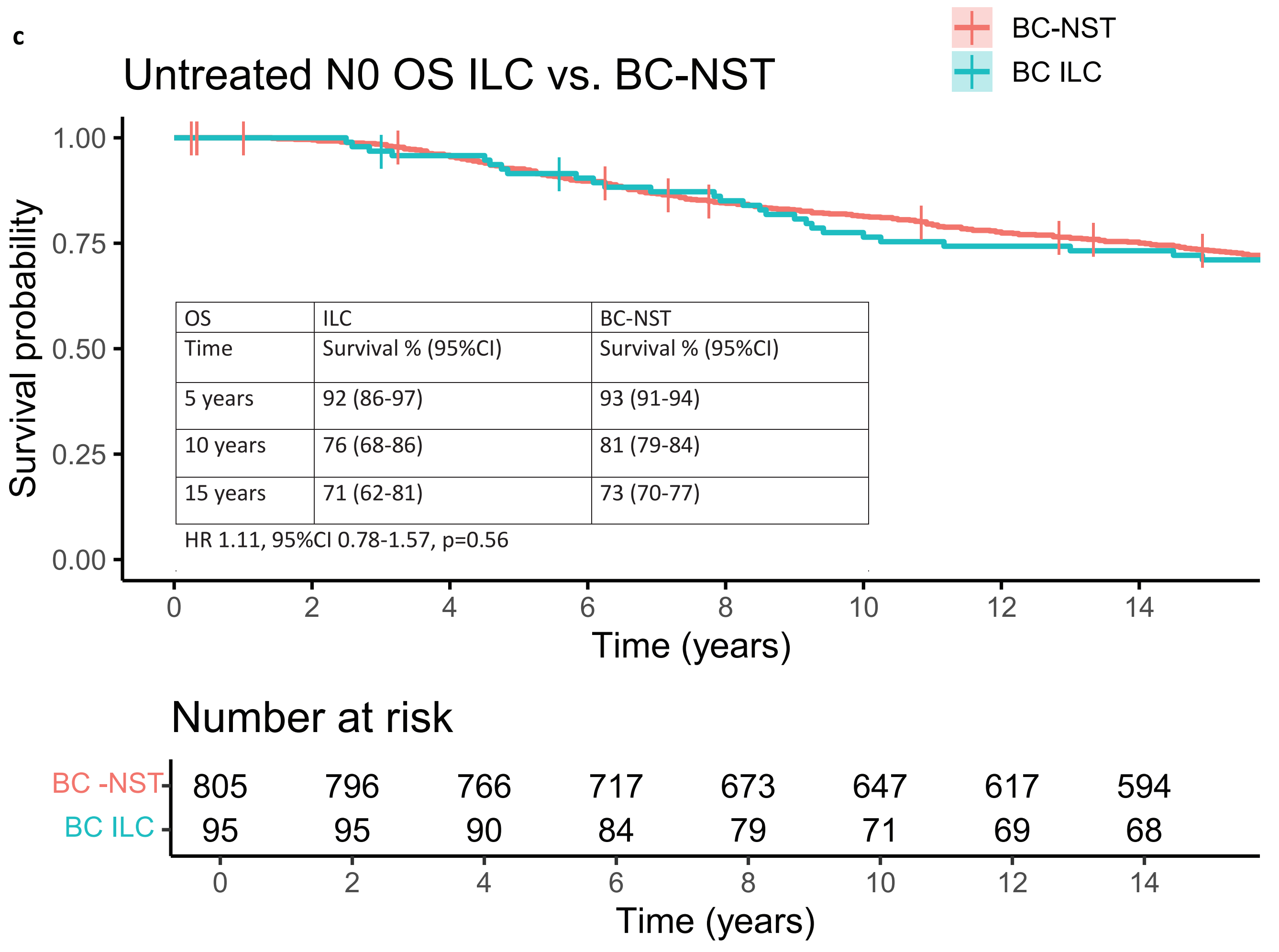

**Supplementary figure 3**

a

RFS low-risk luminal A-like ILC vs. BC-NST

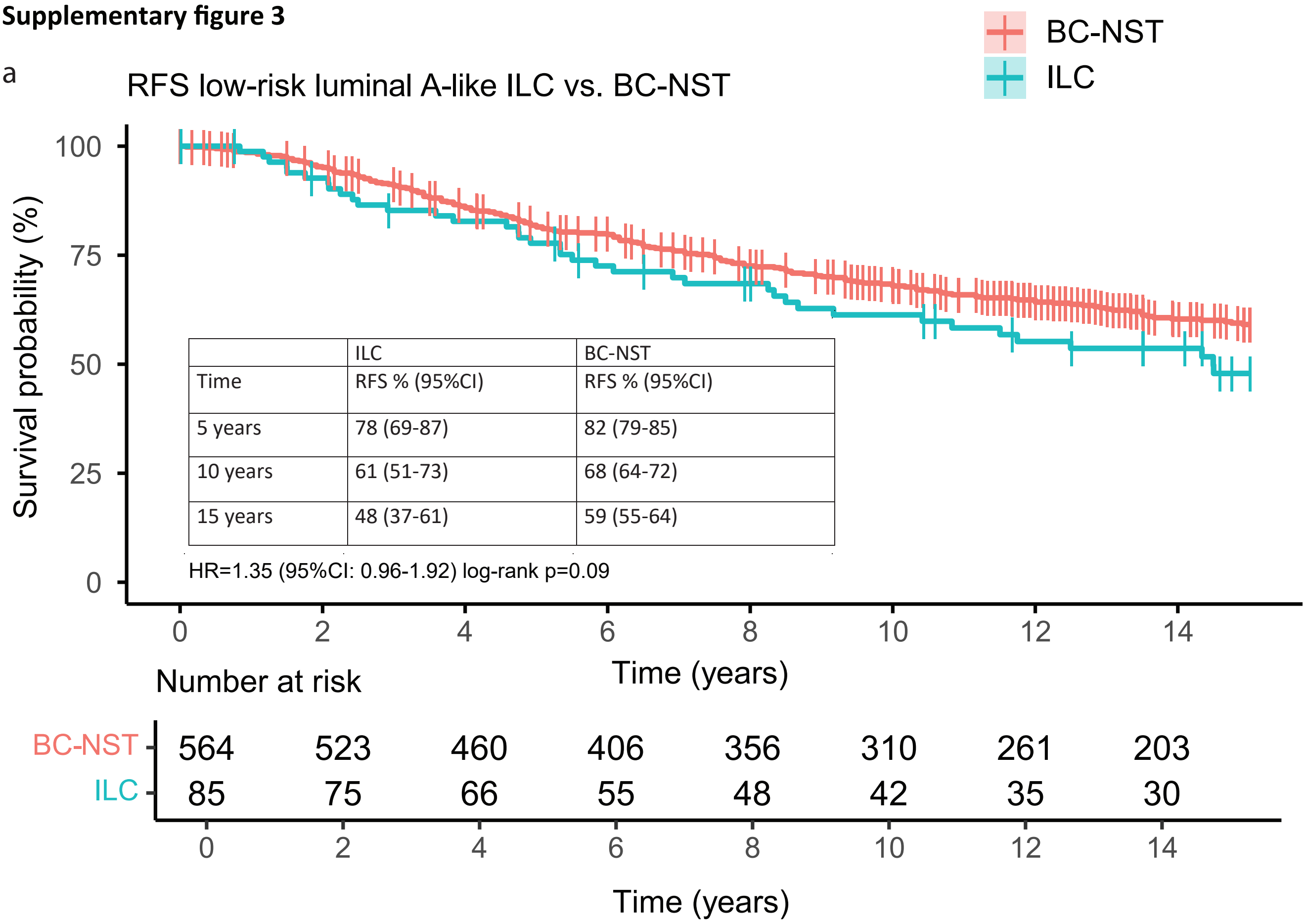


| | ILC | BC-NST |
|---|---|---|
| Time | RFS % (95%CI) | RFS % (95%CI) |
| 5 years | 78 (69-87) | 82 (79-85) |
| 10 years | 61 (51-73) | 68 (64-72) |
| 15 years | 48 (37-61) | 59 (55-64) |

HR=1.35 (95%CI: 0.96-1.92) log-rank p=0.09

| | 0 | 2 | 4 | 6 | 8 | 10 | 12 | 14 |
|---|---|---|---|---|---|---|---|---|
| BC-NST | 564 | 523 | 460 | 406 | 356 | 310 | 261 | 203 |
| ILC | 85 | 75 | 66 | 55 | 48 | 42 | 35 | 30 |

b

OS low-risk luminal A-like ILC vs. BC-NST

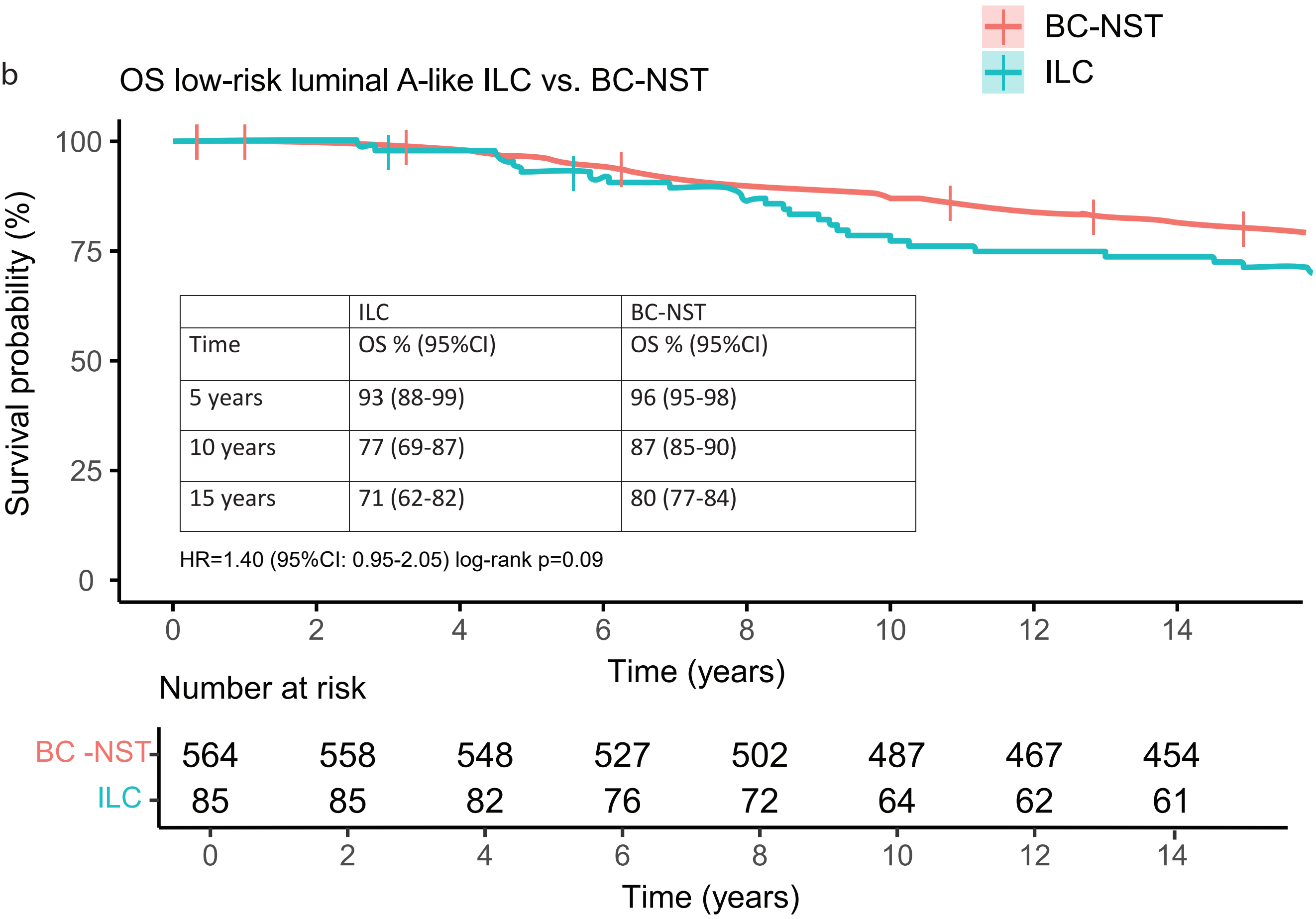


| | ILC | BC-NST |
|---|---|---|
| Time | OS % (95%CI) | OS % (95%CI) |
| 5 years | 93 (88-99) | 96 (95-98) |
| 10 years | 77 (69-87) | 87 (85-90) |
| 15 years | 71 (62-82) | 80 (77-84) |

HR=1.40 (95%CI: 0.95-2.05) log-rank p=0.09

| | 0 | 2 | 4 | 6 | 8 | 10 | 12 | 14 |
|---|---|---|---|---|---|---|---|---|
| BC -NST | 564 | 558 | 548 | 527 | 502 | 487 | 467 | 454 |
| ILC | 85 | 85 | 82 | 76 | 72 | 64 | 62 | 61 |
